# Trafficking and/or division: Distinct roles of nucleoporins based on their location within the nuclear pore complex

**DOI:** 10.1080/15476286.2022.2067711

**Published:** 2022-05-01

**Authors:** Eva Hegedűsová, Veronika Maršalová, Sneha Kulkarni, Zdeněk Paris

**Affiliations:** a Institute of Parasitology, Biology Centre, Czech Academy of Sciences, České Budějovice, Czech Republic; b Faculty of Science, University of South Bohemia, České Budějovice, Czech Republic

**Keywords:** tRNA trafficking, nucleoporins, nuclear division, FG-Nups, NPC, *Trypanosoma brucei*

## Abstract

The nuclear pore complex (NPC) facilitates the trafficking of proteins and RNA between the nucleus and cytoplasm. The role of nucleoporins (Nups) in transport in the context of the NPC is well established, yet their function in tRNA export has not been fully explored. We selected several nucleoporins from different parts of the NPC to investigate their potential role in tRNA trafficking in *Trypanosoma brucei*. We show that while all of the nucleoporins studied are essential for cell viability, only TbNup62 and TbNup53a function in tRNA export. In contrast to homologs in yeast TbNup144 and TbNup158, which are part of the inner and outer ring of the NPC, have no role in nuclear tRNA trafficking. Instead, TbNup144 plays a critical role in nuclear division, highlighting the role of nucleoporins beyond nucleocytoplasmic transport. These results suggest that the location of nucleoporins within the NPC is crucial to maintaining various cellular processes.

## Introduction

Many cellular functions in the eukaryotic cell depend heavily on the exchange of molecules between the nucleus and the cytoplasm; two compartments separated by the nuclear envelope (NE). Nucleocytoplasmic transport occurs through portals called nuclear pore complexes (NPCs), which are embedded in the NE and mediate a continuous flow of various cargoes between the nucleoplasm and cytoplasm. Each NPC consists of approximately 500 individual proteins called nucleoporins (Nups). Multiple copies (from 8 to 64) of around 30 different Nups are arranged in a cylindrical structure with strong octagonal symmetry [1–5]. An NPC’s critical structural elements can be divided into a well-conserved core transport channel, which serves as the conduit for macromolecular exchange, and peripheral components, such as the nuclear basket and cytoplasmic filaments. Nups can be classified into three classes: pore membrane proteins (Poms), containing transmembrane domains that anchor the NPC to the nuclear envelope. The second class includes core scaffold Nups, representing roughly half the mass of the whole NPC. This consists of two inner rings surrounded by two outer rings, providing the platform for anchoring phenylalanine-glycine repeat-containing Nups (FG-Nups). The third class consists of FG-Nup proteins, filling the central channel of the NPC. FG-Nups contain repeats of phenylalanine-glycine motifs separated by hydrophilic spacer sequences (5–30 amino acids), creating a disordered meshwork [2,6]. The NPC is freely permeable to small molecules (~5 nm in diameter) such as metal ions, but constitutes a highly efficient molecular sieve for molecules bigger than 40 kDa. Large macromolecules require the assistance of soluble nuclear transport factors (NTFs), which mainly belong to the karyopherin family. Transport through the central channel of the NPC is mediated by the natively disordered repeat domains of FG-Nups, forming a continually interactive surface for the transport of factor-cargo complex [2]. The interactions between the NTF-FG are weak and transient, which enables cargo to move rapidly through the pore. Approximately a third of all Nups contain FG repeat regions [1,7].

Previously, only the components of NPCs of the yeast *Saccharomyces cerevisiae* and some vertebrates have been characterized in detail [1,2,7], whereas the NPCs of other organisms, including trypanosomatid parasites, remained largely unexplored. The evolutionary origin of the NPC of the highly divergent *Trypanosoma brucei* has only recently been elucidated [8]. Previous studies have revealed that the NPC of this trypanosomatid parasite shares domain organization and compositional similarity on a protein level with opisthokonts, suggesting that basic structural components of the NPC are conserved across the eukaryote domain. However, there are architectural inconsistencies. For example, except within the nuclear basket, the trypanosomal NPC has a complete symmetric localization of nucleoporins [8], in contrast to opisthokonts, where some Nups are asymmetrically organized [9,10]. Compared with the most conserved structural components of other eukaryotes, trypanosomal Nups share very low sequence similarity; only five Nups (TbSec1, TbNup144, TbNup96, TbNup158, and TbNup62) could be easily identified by sequence homology searching [11]. Most glaring is the apparent absence of Mlp/Trp homologs [12–14]. However, two trypanosome nuclear basket proteins TbNup92 and TbNup110 may represent functional analogues of yeast Mlp [11,14].

The primary function of the NPC is nucleocytoplasmic transport. While the mechanism of mRNA export across the NPC has been extensively studied [10,15–18], the export of other classes of RNAs, including tRNAs, still remains to be fully elucidated. Earlier studies have revealed that in yeast, some nucleoporin mutants (*nup49, nup116 and nup145*) show a strong accumulation of unspliced pre-tRNAs [19,20], which represents a hallmark of defective transport due to tRNA splicing being a cytosolic-localized event in yeast [21,22]. The same set of mutants exhibited nuclear retention of poly(A) RNA [15,23]. However, no substantial effects on pre-rRNA processing or the accumulation of mature rRNA were observed [19].

In trypanosomatid parasites, the nuclear-cytoplasmic export factors involved in tRNA trafficking have recently been identified by our group [24]. We showed that the general mRNA exporter TbMex67/TbMtr2 is one of the components of the tRNA export machinery, providing a continuous supply for translation. Unlike in yeast, RNAi downregulation of TbMex67 and TbMtr2 resulted in the accumulation of different subsets of tRNAs in the nucleus of *T. brucei*. While TbMtr2 disrupted the export of all tested tRNAs, silencing of TbMex67 led to the accumulation of tRNAs modified with queuosine, meaning that a subset of tRNAs was transported by a different set of exporter proteins, based on their processing and modification status. In agreement with our previous results, a recent study has revealed that yeast Mex67 may interact with multiple different FG repeat domains (FG, FXFG and GLFG) of Nups in the central channel of the NPC [25].

Although the structure of the NPC has been elucidated and homologs of NPC components recently identified in *T. brucei*, many are yet to be functionally characterized. In this study, we used molecular biological approaches to investigate the role of selected Nups (TbNup62, TbNup53a, TbNup144, and TbNup158) in nucleocytoplasmic transport in *T. brucei*, with a focus on nuclear tRNA export. Our results show that downregulation of the FG-Nups, TbNup62, and TbNup53a affects the nucleocytoplasmic transport of tRNAs. By contrast, nucleoporins TbNup144 and TbNup158, which are part of the inner and outer ring of the NPC, respectively, play no role in tRNA trafficking. Interestingly, the silencing of TbNup144, hampered nuclear division, revealing a function beyond nucleocytoplasmic transport in *T. brucei*. In conclusion, we propose that the role of individual Nups is highly dependent on their location within the NPC.

## Results

### Differentially located Nups are essential for cell viability of T. brucei

**Figure 1.**
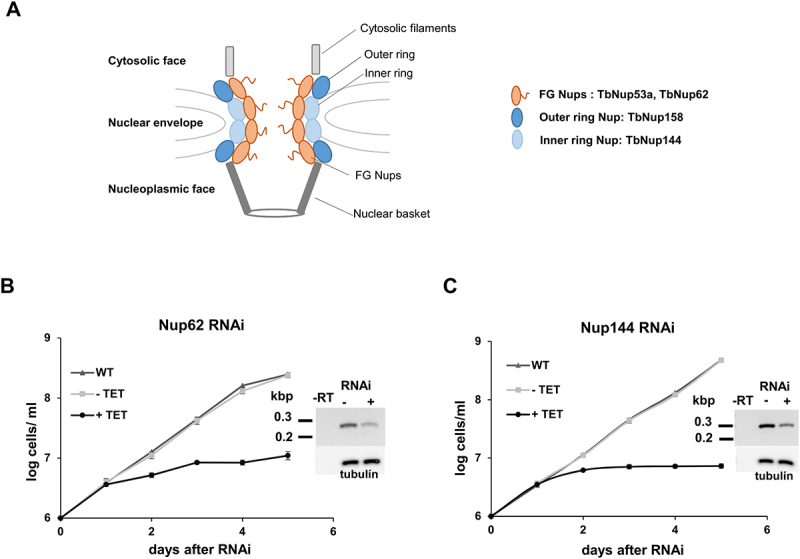
Nucleoporins TbNup62 and TbNup144 are essential for cell survival. (A) Schematic representation of the studied Nups within the NPC. Growth curves of the procyclic form of *T. brucei*, wild-type (WT; triangle), non-induced (-TET; square), and RNAi-induced (+TET; circle) cell lines of (B) TbNup62 and (C) TbNup144. Three biological repeats were performed, and the average of three experiments was plotted. Mean ±SD is shown. Inset: RT-PCR analysis showing the downregulation of the mRNA levels and loading control.

Proteomic approaches (affinity capture purification and mass spectrometry) applied to *T. brucei* have identified approximately 30 nucleoporins, which were assigned as putative yeast and human homologs [8,11]. Based on sequence similarity and predicted location in the nuclear pore complex, as well as their possible role in tRNA nuclear trafficking (shown in yeast), we selected nucleoporins TbNup62, TbNup53a, TbNup144, and TbNup158 for functional analysis (Figure 1A). TbNup62 (Tb927.4.5200) of *T. brucei* was designated as a homolog of both Nup57 (yeast) and NUP54 (human), which belong to the FG-repeat nucleoporins located in the central channel of the NPC. Similarly, FG-Nup TbNup53a (Tb927.11.15560) was identified as a homolog of Nsp1 (yeast) and NUP62 (human). In yeast, Nup57 (homolog of TbNup62) was uncovered by a genome-wide screen as a novel gene product involved in tRNA nuclear-cytoplasmic transport [26]. Moreover, mutations of genes encoding five nuclear pore proteins, including Nsp1 and Nup49 (homologs of TbNup53a and TbNup53b, respectively), caused the accumulation of intron-containing tRNAs in yeast [19,20]. In addition, two other nucleoporins were selected as they represent different locations within the NPC structure compared to FG-Nups. TbNup158 (Tb927.11.980) was found to be a clear homolog of the yeast Nup145 based on sequence conservation [11]. This nucleoporin is part of the outer ring and like its yeast counterpart it also bears FG-repeat motifs. Moreover, a previous study revealed that the yeast mutant *nup145* strongly accumulated unspliced tRNAs [19].

Our last chosen target, TbNup144, is an evolutionarily conserved inner ring subunit. Based on sequence similarity TbNup144 (Tb927.10.8170) was identified as a homolog of Nup157/Nup170 (yeast) and NUP155 (human) [11,27]. These two homologs play an essential role in yeast for NPC formation. However, their depletion did not affect existing NPCs or cell growth [28]. In addition, cells harbouring a mutation of the gene encoding Nup170 accumulate intron-containing tRNA^Ile^ [26]. The subcellular localization of the selected Nups in the nuclear envelope of *T. brucei*, has been previously shown by DeGrasse and colleagues and/or TrypTag.DB [11,29].

To investigate the potential function of these Nups (Figure 1A) in tRNA nuclear export within the procyclic form *T. brucei*, we constructed RNAi-inducible cell lines by cloning a portion of the open reading frame into a tetracycline-inducible p2T7-177 RNAi vector. Downregulation of expression of each gene was confirmed by RT-PCR (Figure 1B & 1C; Supplementary Fig. S1A). RNAi cell lines were grown in the presence (+TET) or absence (-TET) of tetracycline. Knock-downs of TbNup62, TbNup53a, TbNup144, and TbNup158 resulted in a severe growth defect (Figure 1B & 1C, Supplementary Fig. S1A & S2A), leading to the conclusion that all four proteins are essential for cell viability.

### FG-Nups are critical for nuclear tRNA trafficking

**Figure 2.**
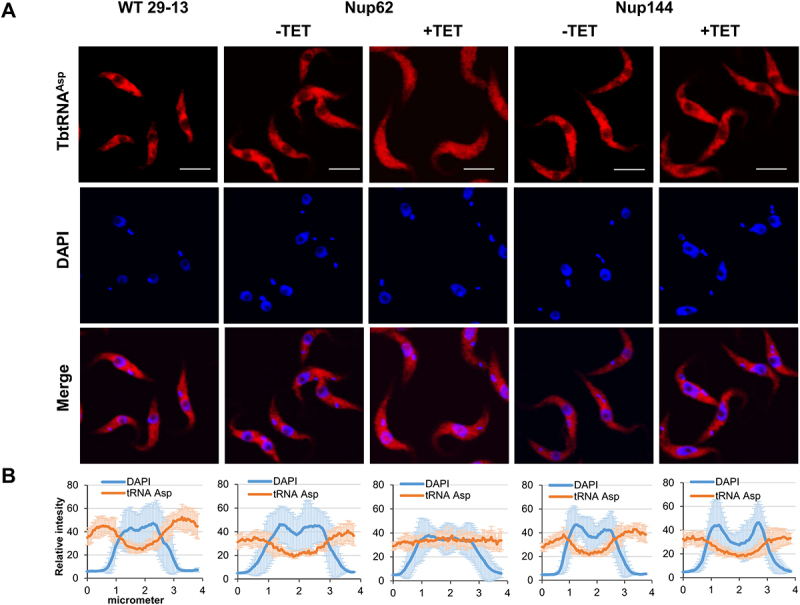
Downregulation of nucleoporin TbNup62 affects nuclear export of tRNA^Asp^, while TbNup144 does not affect tRNA^Asp^ trafficking. (A) To determine the subcellular localization of mature tRNA^Asp^ in WT, non-induced (-TET) and RNAi-induced (+TET) TbNup62 and TbNup144 cells fluorescent *in situ* hybridization was performed. Micrographs show the subcellular localization of mature tRNA^Asp^ (red-Cy3). DAPI (blue) was used to stain the kinetoplast and nucleus DNA. Bars, 5 µm. (B) Quantification of the fluorescence intensity of tRNAs (orange) and DAPI (blue) in non-induced and 28 hr RNAi-induced TbNup62 and TbNup144 cell lines. Each graph shows the intensity profile of individual fluorophores (orange-tRNA^Asp^, blue-DNA) of 6 randomly selected cells, representing the relative intensity average ±SD.

**Figure 3.**
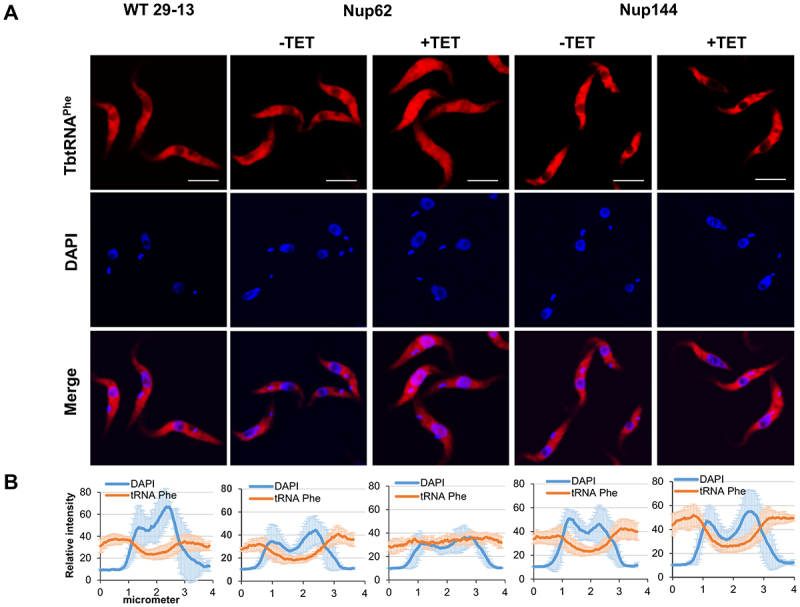
Downregulation of nucleoporin TbNup62 affects nuclear export of tRNA^Phe^, while TbNup144 does not affect tRNA^Phe^ trafficking. (A) To determine the subcellular localization of mature tRNA^Phe^ in WT, non-induced (-TET) and RNAi-induced (+TET) TbNup62 and TbNup144 cells fluorescent *in situ* hybridization was performed. Micrographs show the subcellular localization of mature tRNA^Phe^ (red-Cy3). DAPI (blue) was used to stain the kinetoplast and nucleus DNA. Bars, 5 µm. (B) Quantification of the fluorescence intensity of tRNAs (orange) and DAPI (blue) in non-induced and 28 hr RNAi-induced TbNup62 and TbNup144 cell lines. Each graph shows the intensity profile of individual fluorophores (orange-tRNA^Phe^, blue-DNA) of 6 randomly selected cells, representing the relative intensity average ±SD.

To investigate the subcellular distribution of tRNAs in generated knock-downs, we performed fluorescent *in situ* hybridization (FISH). Our previous report showed that the nuclear export factor TbMex67/TbMtr2 plays an essential role in tRNA trafficking. However, we found that TbMex67 only impacted the nuclear export of tRNAs that can be modified with queuosine (Q), a tRNA modification attached in the nucleus. By contrast, trafficking of other tRNAs (non-Q containing) was not affected [24]. Based on our previous experience, we selected two tRNAs for the current analysis. tRNA^Asp^ contains GUN anticodon and thus can be modified with queuosine (Q) at wobble position 34 in tRNAs and tRNA^Phe^, which is naturally devoid of Q modification. FISH analysis revealed that the pool of tRNA^Asp^ and tRNA^Phe^ was increased in the nucleus in the TbNup62 RNAi-induced cell line compared with uninduced and control cells (WT), where the nucleus remains empty (Figure 2A & 3A). The same outcome was achieved by the downregulation of TbNup53a (Supplementary Fig. S1C). FISH also indicated that TbNup62 and TbNup53a do not discriminate between Q-containing and Q-free tRNAs. Downregulation of TbNup144 (Figure 2A & 3A) and TbNup158 (Supplementary Fig. S2C) caused no significant changes in tRNA localization, as both tRNAs were distributed predominantly to the cytoplasm in all of the tested cell lines (induced, uninduced, and WT control) with no nuclear accumulation. To quantify the tRNA subcellular distribution of induced vs. uninduced cell lines, we plotted the fluorescence intensities of the FISH and DAPI signal. The maximum FISH signal for tRNA^Asp^ and tRNA^Phe^ co-localized with the DAPI signal in the TbNup62 (Figure 2B & 3B) and TbNup53a (Supplementary Fig. S1D) RNAi cell lines, while the FISH signal of the same tRNAs showed no overlap with the DAPI signal in the TbNup144 (Figure 2B & 3B) and TbNup158 RNAi cell lines (Supplementary Fig. S2D).

**Figure 4.**
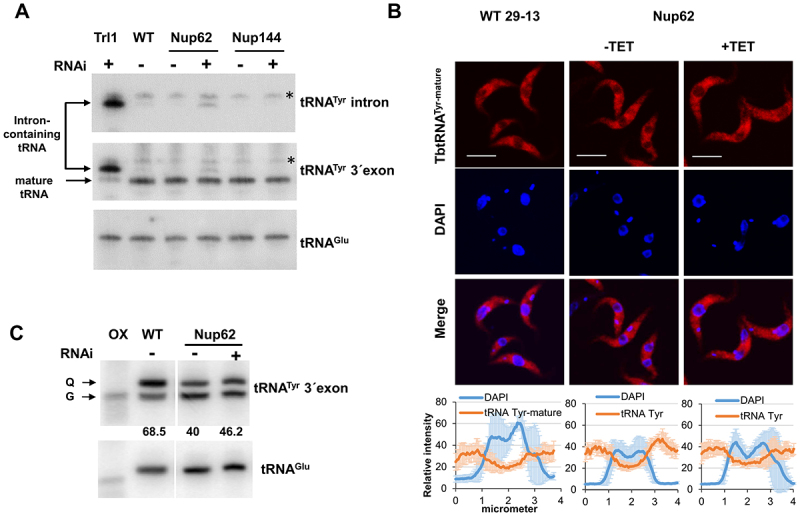
The TbNup62 has role in the primary export of intron-containing tRNA, while subcellular localization of mature tRNA^Tyr^ is not affected (A) Total RNA was isolated from wild-type (WT), non-induced (-TET), and RNAi-induced (+TET) cells (TbNup62, TbNup144), and Northern hybridization was performed with a tRNA^Tyr^-intron and tRNA^Tyr^-3’ exon probe to detect intron-containing and/or mature tRNA. Trl1 RNAi-induced cells were used as a positive control for detection of intron-containing tRNA^Tyr^. tRNA^Glu^ was used as a loading control for the experiment. The blots are representative of three independent experiments. The higher faint band, which is present in all cell lines tested, is a result of unspecific hybridization (marked with an asterisk). (B) To determine the subcellular localization of mature tRNA^Tyr^ in WT, non-induced (-TET) and RNAi-induced (+TET) TbNup62 cells fluorescent *in situ* hybridization was performed. Micrographs show the subcellular localization of mature tRNA^Tyr^ (red-Cy3). DAPI (blue) was used to stain the kinetoplast and nucleus DNA. Bars, 5 µm. Panel below the micrographs shows quantification of the fluorescence intensity of tRNAs (orange) and DAPI (blue) in non-induced and 28 hr RNAi-induced TbNup62 cell line. The graphs under each column show the intensity profile of individual fluorophores (orange-tRNA^Tyr^, blue-DNA) of 6 randomly selected cells of that cell line, where the values represent the relative intensity average ±SD. (C) Total RNA was collected after 28 hr of RNAi induction. Boronate affinity electrophoresis – Northern blotting was carried out for RNAi-induced (+TET) and non-induced (-TET) cells. Probes for tRNA^Tyr^ 3’ exon were used to determine Q levels. ‘ox’ refers to an oxidized control; oxidation of the cis-diols from Q prevents the observed band shift. tRNA^Glu^ probe was used as a loading control. Numbers under the blot indicate % Q tRNA modification levels, compared to total tRNA^Tyr^ levels.

Based on the FISH data, we focused on intron-containing tRNA as a next step. Like in yeast, tRNA splicing in *T. brucei* is a cytoplasmic event [30]. As a result, tRNA^Tyr^, the only intron-containing tRNA in *T. brucei*, needs to be exported through the NPC following transcription to be spliced. To test if the downregulation of either of the Nups mentioned above causes nuclear accumulation of the unspliced tRNA, we performed Northern hybridizations with an intron-specific probe for tRNA^Tyr^ in our RNAi cell lines. The tRNA ligase (Trl1) RNAi cell line [30], in which unspliced tRNA accumulates, was used as a positive control to detect intron-containing tRNA^Tyr^. tRNA^Glu^ served as a loading control. We observed an enrichment of intron-containing tRNA^Tyr^ after downregulation of TbNup62 (Figure 4A) as well as TbNup53a (Supplementary Fig. S1B), but not in the TbNup144 (Figure 4A) and TbNup158 RNAi-induced cell lines (Supplementary Fig. S2B). Therefore, our results indicate that FG-nucleoporins located in the central channel of the NPC can affect the levels of unspliced tRNA, either through trafficking delays or as an indirect effect of the defective splicing machinery. This result prompted a question. If the nuclear export of intron-containing tRNA is affected, will the distribution of mature tRNA^Tyr^ also be altered?

Following cytoplasmic splicing, mature spliced tRNA^Tyr^ needs to be imported back to the nucleus to obtain Q modification [31]. This fact, combined with our observation of intron-containing tRNA detection after downregulation of TbNup62 (Figure 4A), led us to speculate that the transport of mature tRNA^Tyr^ and its Q modification status, might also be affected. To test this hypothesis, we performed FISH to assess the subcellular localization of mature tRNA^Tyr^. We also determined the levels of the Q modification in this tRNA using boronate affinity gel electrophoresis. As described previously, this method is based on the affinity of *cis*-diol groups to 3-(acrylamido) phenylboronic acid (APB), resulting in a differential electrophoretic mobility between Q-tRNA and their unmodified counterparts [32]. Additionally, tRNA^Glu^, which is not a substrate for Q modification, was used as a loading control. Since Q modification is a nuclear event, nuclear accumulation of tRNA^Tyr^, leads to an increased Q-tRNA^Tyr^ level, a phenomenon described previously [24]. However, the FISH result did not show any significant change in the subcellular distribution of mature tRNA^Tyr^ in the TbNup62-induced cell line (Figure 4B). The APB gel-Northern blot for detection of Q also manifested no increase in Q-tRNA^Tyr^ modification (Figure 4C), suggesting that trafficking of the mature tRNA^Tyr^ might require a specialized export pathway.

Although no general impact on tRNA trafficking was observed upon TbNup144 and TbNup158 depletion, the substantial growth defect of the TbNup144 RNAi-induced cell line (Figure 1C) suggests its importance in *T. brucei*, unrelated to tRNA trafficking. Since TbNup53a shows the same phenotypic pattern as TbNup62, we assume that FG-nucleoporins located inside the central core might have overlapping roles within the NPC. TbNup158, described as an outer ring nucleoporin with FG-repeat motifs, did not change the subcellular localization of the tested tRNAs.

### TbNup144 has a role in nuclear division

**Figure 5.**
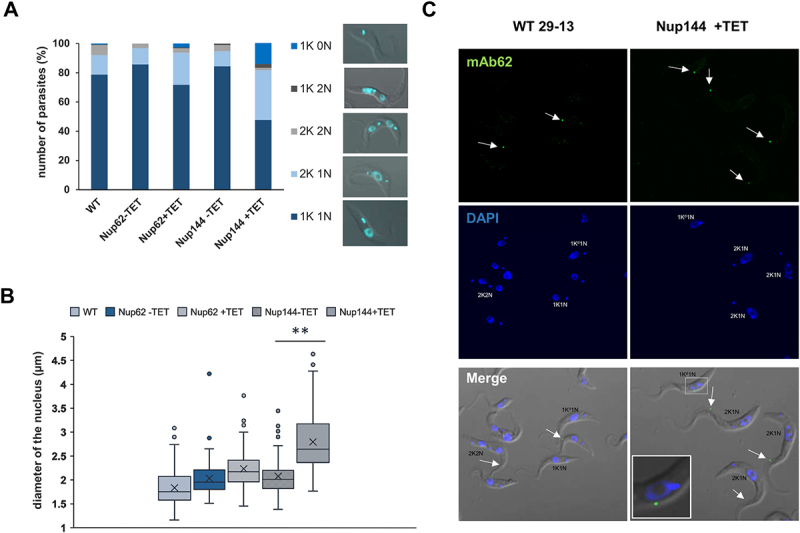
Downregulation of nucleoporin TbNup144 causes a severe defect in nuclear division. (A) Quantification of nuclear (N) kinetoplast (K) ratio in parental (WT), non-induced (-TET) and 28 hr RNAi-induced (+TET) TbNup62 and TbNup144 cells. 300 cells from each condition were counted to categorize the distinct stages of the cell cycle. TbNup144 RNAi-induced cells show an increased number of 2K1N cell types, suggesting arrest in the G2/M phase. Right panel: Example specimen of procyclic form of *T. brucei* during different cell cycle stages with the different kinetoplast (K) and nucleus (N) content. 1K0N and 1K2N are cell types with aberrant cytokinesis. (B) Determination of the length of the nucleus. 100 cells from each cell line were used to measure the length of the nucleus from the posterior to the anterior pole of the nucleus. Downregulated TbNup144 (+TET) shows a significant change in the size of the nucleus compared with un-induced (-TET) or parental cell line (** p < 0.0001). (C) Representative micrographs showing the 2F2K1N cell type after 28 h RNAi-induction of TbNup144 cell line. To determine the cell cycle phase of WT and RNAi-induced TbNup144 cells, immunofluorescence was performed. Antibody mAb62 (green) detecting the flagella connector on the tip of the new flagella and DAPI (blue) staining showing the differences of kinetoplast and nucleus content between parental (WT) and RNAi-induced TbNup144 cells. Arrows point to the cells containing new and old flagella.

During the analysis of the subcellular distribution of tRNAs, we observed an increased ratio of kinetoplasts (mitochondrial DNA of *T. brucei*) to nuclei and an elongation of the nucleus after TbNup144 RNAi. The cell cycle defect caused by ablation of TbNup144 was analysed by DAPI staining to visualize nuclear (N) and kinetoplast (K) DNA. A 3.4-fold increase (from 10% to 34%) of the 2K1N type of cells was observed after TbNup144 knock-down. Another prominent phenotype was a 14-fold increase in the formation of zoids or anucleated cells (1K0N), indicating a failure of nuclear division in the absence of TbNup144. The defect in nuclei segregation also manifested in a 2.7-fold decrease in the numbers of 2K2N type of cells in the RNAi-induced cell line compared with controls (Figure 5A).

Next, we measured the diameter of the nucleus in our induced and uninduced cell lines. In TbNup144 RNAi-induced cells, elongated nuclei were in abundance. To quantify this size difference, the diameter of the nucleus of 100 randomly selected cells (from the anterior to the posterior pole of the nuclei) was measured. In control cells, the average diameter of the nucleus was ~2 µm, whereas, in RNAi-induced cells, the length was up to 4 µm (Figure 5B), potentially corresponding to the nuclear state before karyokinesis (anaphase). This supports our previous observation. We noticed that approx. 30% of the total cell population contained a nucleus longer than 3 µm in TbNup144 RNAi-induced cells, compared with the uninduced or WT controls, where the percentage was between 1 and 5%.

Prior to nuclei division, the kinetoplast and basal body need to be duplicated before a new flagellum is formed. Since DAPI staining showed that kinetoplast was able to divide in these cells, we investigated the subsequent step in the cell cycle, which is the formation of a new flagellum. In TbNup144 RNAi-induced cells, we performed an immunofluorescence assay with the antibody mAb62, recognizing the flagella connector between old and new flagella [33]. Normally, in the exponentially growing culture, 1F1K1N cells constitute the largest morphological category (Figure 5A). However, our result revealed (Figure 5C), that the downregulation of TbNup144 led to an increased number of cells with two flagella, two kinetoplasts but only one nucleus (2F2K1N) compared with control cells (WT). This suggests that events up to the duplication of the kinetoplast and the flagellum are not affected, and that the cells are likely arrested at this specific point during the cell cycle.

**Figure 6.**
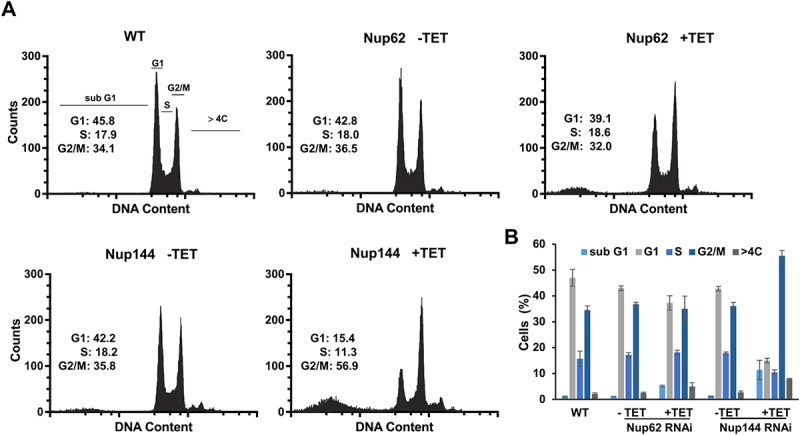
Silencing of TbNup144 but not of TbNup62 causes cell cycle arrest in the G2/M phase. (A) Flow cytometry profiles of parental (WT), non-induced, and RNAi-induced TbNup62 and TbNup144 cells. To determine the karyotype of the population, cells were fixed and stained with propidium iodide (indicating DNA content) (*x*-axes). Cell count is plotted on the *y*-axes. Downregulation of TbNup144 shows arrest in the G2/M phase (2K1N), suggesting that cell lines after induction encounter errors in completing mitosis and cytokinesis. Three biological repeats were performed, and representative histograms are shown. Inset: Percent of cells in G1, S and G2/M are shown. (B) Densitometry analysis of the cell cycle phases (sub-G1, G1, S, G2/M, >4C). Mean scores from three biological replicates are plotted, and ±SD is shown.

For further confirmation, we employed flow cytometry analysis to detect the potential changes in the cell cycle of the TbNup144 RNAi cell line. As the histograms of propidium iodide staining show, after 28 h of induction of the TbNup144 RNAi cell line, cells were arrested in the G2/M checkpoint (Figure 6A). The percentage of the TbNup144 RNAi cells in the G2/M phase of the cell cycle after induction was increased from 35 to 56%, and at the same time, the number of cells in the G1 phase dropped from 42% to 15% (Figure 6A & 6B). In addition, an increased number (average 11.3%) of cells in the sub-G1 phase of induced cells was also detected. These results indicate that downregulation of TbNup144 causes defects in nuclear division. As mentioned above, depletion of TbNup144 resulted in an increased number of cells with two kinetoplasts and one nucleus (Figure 5A). This cell type (2K1N) is present in normal cells during the division of *T. brucei* from S phase to metaphase (or early anaphase) as regular steps of the cell cycle. However, the following steps, such as telophase (2K2N) and later cytokinesis were not observed in the TbNup144 RNAi cell line.

The other selected nucleoporins (TbNup62, TbNup53a, and TbNup158) were also tested for their role in nuclear division since increased numbers of 2K1N cells were present in all knock-down cell lines. However, for TbNup62 (Figure 5A), TbNup53a, and TbNup158 (Supplementary Fig. S3A) the effect was less pronounced (approx. 2.0 fold). Accordingly, flow cytometry performed on the TbNup62 (Figure 6A & 6B) and TbNup53a (Supplementary Fig. S3 B&C) RNAi cell lines exhibited only minor changes in the cell cycles compared with TbNup144 RNAi (Figure 6A & 6B). In the case of the outer ring of TbNup158, RNAi induction led to an increase in the proportion of cells in the sub-G1 phase. Nevertheless, the effect on the ratio between G1, S, and G2/M seems not to be as pronounced (Supplementary Fig. S3B) when compared to TbNup144 (Figure 6B). Since a significant increase of cells of the sub-G1 phase was detected in the induced TbNup144 and TbNup158 RNAi cell lines, we performed annexin/PI staining to distinguish between early or late apoptotic cells (or dead cells). The presence of annexin positive but PI negative cells in both tested cell lines indicated populations of early apoptotic cells, but not dead cells. We hypothesize that the sub-G1 population was represented in this cluster. TbNup144 RNAi cells contain around 11% of cells in the early apoptotic stage, compared with TbNup158 (3,8%), which is only slightly higher than the control (2,5%) (Supplementary Fig. S4).

These results indicate that TbNup144, but not TbNup62 or TbNup53a, is necessary for regular segregation of the nucleus, but its exact role during karyokinesis remains unknown. In the case of TbNup158, the effect on the cell cycle was somewhat intermediate. Although the relative ratio of G1 and G2/M phases was similar to that observed in TbNup62 and TbNup53a RNAi cell lines, we did notice an enrichment in the sub-G1 phase (Supplementary Fig. S3 B&C). These were most likely the zoid cells (1K0N) (Supplementary Fig. S3A), also observed by microscopy. However, the population of annexin-positive cells was significantly lower in the TbNup158 RNAi cell line than in the TbNup144 (Supplementary Fig. S4). This suggests that the effect of TbNup158 on cell cycle progression was considerably delayed or indirect, concurrent with the delayed growth defect observed in this cell line. The impact of silencing FG-Nups on the cell cycle was minimal compared to TbNup144, indicating that this is not an incidental effect of RNAi or a more global destabilization of the NPC. A more comprehensive investigation is needed to better understand the detailed molecular mechanism behind the role of TbNup144 in nuclear division, which may be a key point in the cell cycle regulation of these medically important parasites.

## Discussion

Over the past few decades, the nuclear pore complex has been studied in various model systems to understand its structure and function [1,2,7]. The morphology of this complex structure appears to exhibit conserved features throughout eukaryotes [11]. The nucleocytoplasmic trafficking of the macromolecules is facilitated by interactions between nucleoporins located inside the central channel as well as the transport factor-cargo complex. In addition to the more established roles of NUPs in the transport process, several alternative roles of nucleoporins have been revealed over the years. Overall, our results support the conserved structure and function of NPC, but also suggest differences in function between some *T. brucei* nucleoporin homologs compared with other model organisms.

This study explored the possibility of nucleoporins localized within different regions of the NPC in *T. brucei* having distinct cellular functions. This hypothesis is based on a previous report showing that the NPC central channel is filled with FG-Nups, which provide the interface for transient interactions with the transport factor-cargo complex. By downregulation of nucleoporins located in this channel, the functionality of the NPC can be disturbed at the level of nucleocytoplasmic transport. To test our hypothesis, we selected four candidates known to play a role in pre-tRNA splicing in yeast. Interestingly, only two of the candidates, TbNup62 and TbNup53a (homologs of yeast Nup57 and Nsp1), affected unspliced tRNA levels. However, TbNup144 and TbNup158 showed no similar phenotype, contrary to their yeast homologs Nup170 and Nup145.

tRNA splicing is a cytoplasmic event in *T. brucei*. As described previously, after primary processing (5’ and 3’ end processing), tRNA is exported to the cytosol for splicing [30]. We attempted to detect the change in abundance of intron-containing tRNA^Tyr^ after depletion of selected Nups. The silencing of TbNup62 and TbNup53a, which both belong to the FG-Nups, caused a detectable accumulation of unspliced tRNA, indicating their possible role in nuclear tRNA export. Importantly, we cannot rule out the possibility that the observed intron accumulation represents a secondary effect of tRNA splicing defects caused by a general export blockage.

Furthermore, we tested the subcellular localization of mature tRNAs by FISH. Depletion of TbNup62 and TbNup53a resulted in the nuclear accumulation of tRNA^Asp^ and tRNA^Phe^. However, no significant nuclear accumulation of mature tRNA^Tyr^ was detected. The possible explanations are either: 1) the primary export of intron-containing tRNA^Tyr^ is slowed down, but not to an extent that affects mature tRNA^Tyr^ distribution; 2) the retrograde import delivering the spliced tRNA^Tyr^ back to the nucleus might be altered due to TbNup62 depletion. Since Q formation is a nuclear event, by blocking retrograde transport spliced tRNA^Tyr^ cannot be delivered back to the nucleus to obtain Q modification. In such a scenario, cells can contain only a certain amount of modified tRNA^Tyr^, which was present before the nucleocytoplasmic transport was inhibited or 3) the overall effect on the kinetics of retrograde import as re-export of mature tRNA^Tyr^ is minimal. It is difficult to resolve which of these (or any other) possibilities is true, as we are limited to a short-time frame (28 hrs) of study due to the severe growth defect caused by the downregulation of TbNup62. Compared with tRNA^Tyr,^ the situation of the other two tested tRNAs (tRNA^Asp^, tRNA^Phe^) is different, since only tRNA^Tyr^ needs to undergo retrograde transport to obtain the Q modification, meaning that the observed retention of these other tRNAs is presumably affected at the level of initial primary export.

These results indicate that TbNup62 and TbNup53a ablation likely disturbs interactions between FG-Nups and the export receptors-cargo complex, ultimately disrupting trafficking across the nuclear pore. Although many different FG-Nups are part of the core transport channel (Nup62, Nup53a and Nup53b), our results prove that even one ablated FG-Nup can affect tRNA export. By contrast, downregulation of NPC ring-constituting Nups (TbNup144 and TbNup158), does not cause tRNA accumulation, unlike in their yeast counterparts, Nup170 and Nup145. Two scenarios can explain this. First, nucleoporins located in the inner ring do not directly interact with the export receptor-cargo complex; therefore, the transport channel can fulfill the exchange between nucleus and cytosol without them. This agrees with data from Obado and colleagues, who, using affinity purification, demonstrated that TbNup144 interacts only with TbNup89 (part of the outer ring) and not with other, inner-ring, proteins [8]. In contrast, the yeast homolog (Nup170/Nup157) interacts with other inner-ring, outer-ring, and pore-membrane Nups (Nic96, Nup53, Nup59, etc.) [9]. The second explanation is that the lack of TbNup144 does not destabilize the structure of the NPC central channel to the extent that affects the transport processes required by the cell. Although TbNup144 does not have a role in nuclear tRNA export, its RNAi silencing causes a severe proliferative defect supporting the essential role of TbNup144 in trypanosomes. In the case of TbNup158, the presence of the FG repeat motif led us to assume that its role in transport is similar to that of TbNup62 and TbNup53a. However, we did not observe a change in the subcellular localization of any tested tRNAs.

To complete cell division, eukaryotic cells must precisely replicate and faithfully segregate all organelles (together with their genetic material) to the daughter cells. Therefore, eukaryotes employ several cell-cycle checkpoints to ensure the fidelity of cell division. The different stages of the cell cycle in *T. brucei* can be distinguished by the numbers of nuclei and kinetoplasts within each cell. In the G1 phase, the cell contains a single basal body that sits near the base of the flagellum and associates with a single kinetoplast. Cells in the S phase duplicate the basal body and kinetoplast. However, the kinetoplast is not yet physically separated [34]. First, the kinetoplast divides during the G2 phase, while the nucleus remains undivided (2K1N). In the subsequent cell cycle phase (mitosis), the nucleus is segregated (2K2N), and the cell cycle is finished with cytokinesis, whereas during normal circumstances two nuclei and two kinetoplasts are equally separated to each of the daughter cells (1K1N).

In most cases, proper segregation of the nucleus is mandatory before cytokinesis. However, there are instances where cell division can occur in the absence of mitosis, such as in the RNAi-knockdown of mitotic cyclin/CYC2/CRK or after chemical intervention [35–38]. Equally, the procyclic form of trypanosomes is still capable of completing its cytokinesis, leading to the production of zoids (anucleate cells) [38]. This suggests that the procyclic forms lack a checkpoint system for controlling mitosis-cytokinesis transition, or that the mitotically defective procyclic trypanosomes can bypass the mitosis-cytokinesis checkpoint [39]. After depletion of TbNup144, we found an increased number of 2K1N cells with elongated nuclei.

In contrast, the amount of 2K2N cell type significantly decreased, indicating that most cells cannot pass through the last phases of mitosis (anaphase and telophase) and initiate cytokinesis. Additionally, abnormal 1K0N and 1K2N (small proportion) types of cells were observed, suggesting aberrant cytokinesis, producing either multinucleate (2 nuclei) or anucleate cells containing one kinetoplast. We note that multinucleate cells were rare compared with anucleate cells. Indeed, the results from cell cycle analysis of TbNup144 RNAi-induced cells showed that cells are arrested in the G2/M checkpoint. The presence of an elongated nucleus within the cell suggests that the cells had difficulties passing through anaphase, when the spindle fibres begin to pull the chromosomes (via their kinetochores) to opposite poles. In *T. brucei*, more than 200 chromosomes/DNA elements must be segregated during mitosis. However, only a few kinetochores have been observed, suggesting unusual chromosome segregation mechanisms [40–43]. The role of TbNup144 in nuclear division remains a question. Although NPC functions primarily in nucleocytoplasmic transport, experiments over the years have shown that NPCs are linked with many different cellular processes, including mitotic events, cell cycle progression, chromatin organization, kinetochore assembly, spindle checkpoint control, and cytokinesis [44–53]. NPC is not a static structure [54], and individual nucleoporins can be very dynamic, with their turnover in NPCs ranging from seconds to days [55,56]. In contrast to higher eukaryotes, trypanosomes and yeasts undergo closed mitosis. In this process, the nuclear envelope remains intact throughout mitosis, and the mitotic spindle is anchored to the nuclear envelope at the opposite ends of the nucleus [57,58]. It has been shown that some Nups have a role in kinetochore function and mitosis [49,50,52,59,60] and several Nup-chromatin interactions occur within the nuclear interior and away from the NPC itself.

Interestingly, the role of nucleoporins in spindle associations and chromosome segregation has also been described in *T. brucei* [11,14]. These studies revealed that the nuclear basket protein TbNup92 exhibits cell cycle-dependent localization. Whereas most *T. brucei* nucleoporins remain associated within the NPC throughout mitosis, TbNup92 relocates during late mitosis to the NE regions similar to the spindle attachment site. Interestingly, TbNup92 knock-down cell lines do not show a significant proliferative defect, although the accumulation of 1K1N cells after TbNup92 knock-down suggests delays in progression through the G1/S phase. Our observations indicate that TbNup144 RNAi-induced cells are arrested in the G2/M checkpoint.

There are other reported examples of connections between nucleoporins and cell division. In *S. cerevisiae*, NUP170 encodes a specialized nucleoporin with a unique role in chromosome segregation and possibly kinetochore function [61]. Additionally, Nup157 (a paralog of Nup170) has nucleic acid-binding activity, showing additional evidence of physical links between nucleoporin and chromatin [62]. In yeast, it is known that the spindle assembly checkpoint monitors for microtubule-kinetochore attachment errors [63–68]. When an attachment error is detected, anaphase is delayed. As we demonstrated, RNAi-induced cells of TbNup144 encounter difficulties overcoming late anaphase. The established conservation between TbNup144 and its yeast homologs Nup157 and Nup170 and our new results indicate the possible role of TbNup144 in chromosome segregation or kinetochore function within Trypanosomes.

In conclusion, while nucleoporins belonging to FG-Nups (TbNup62, TbNup53a) located in the central channel affect tRNA trafficking, structural nucleoporins TbNup144, and to some extent TbNup158, appear to have a role in nuclear segregation, ultimately causing defects in cell division. Thus, our results highlight that nucleoporins located in different parts of the NPC can influence distinct cellular functions.

## Material and Methods

### Cell culture and generation of cell lines

The procyclic form of *T. brucei* strain 29–13 was grown at 27°C in SDM-79 media supplemented with 10% foetal bovine serum, containing hygromycin (50 µg/ml) and geneticin (15 µg/ml). RNA interference (RNAi) constructs were generated by cloning a portion of the coding sequence of TbNup62 (size of insert 730 bp), TbNup53a (500 bp), TbNup144 (984 bp), and TbNup158 (1644 bp) into plasmid vector p2T7-177 [69]. All inserts for the plasmid were generated by PCR from the genomic DNA of *T. brucei* 29–13 using oligonucleotides (see Supplementary Table S1).

Cloning sites for HindIII and BamHI are underlined in the sequence. Final plasmids were linearized by NotI and transfected into *T. brucei* for genomic integration. Transfectants were selected with phleomycin (2.5 µg/ml). RNAi was induced with 1 µg/ml of tetracycline. Cell density was measured every 24 hours using the Beckman Coulter Z2 counter.

### RT-PCR analysis

Reverse transcription was carried out with total RNA, as described by the manufacturer (QuantiTect Reverse Transcription Kit, Qiagen). The resulting cDNA was PCR amplified with corresponding oligonucleotide primers (Supplementary Table S2).

### Denaturing gel electrophoresis and Northern hybridization

Total RNA was isolated using the guanidinium isothiocyanate/phenol/chloroform extraction method as previously described [70] 5 µg of RNA was resolved by denaturing gel electrophoresis (8% acrylamide, 7 M urea), electroblotted to Zeta probe®(Bio-Rad) membranes, and UV cross-linked (1200 µJoules x100). The membranes were probed with oligonucleotides radiolabeled with ©[32]P-dATP (Supplementary Table S3). Northern hybridization was performed according to the manufacturer’s instructions (Bio-Rad). Subsequently, the membranes were exposed overnight to a Phosphorimager screen and analysed using Typhoon^TM^ 9410 scanner and ImageQuant TL software (GE Healthcare). Boronate affinity electrophoresis was performed as previously described [31,32]. The membranes were probed with oligonucleotides radiolabeled with ©[32]P-dATP: tRNA^Tyr^ 3ʹexon and tRNA^Glu^.

### Fluorescence in situ hybridization (FISH)

1 × 10^7^ cells were harvested and washed with PBS. Cells were resuspended in 4% paraformaldehyde/PBS solution and fixed to poly-L-lysine coated microscope slides for 30 min. Non-adherent cells were removed by washing with PBS, and the remaining cells were dehydrated by a series of increasing ethanol concentrations (50,80 and 100%, for 3 min each). Subsequently, the permeabilized cells were pre-hybridized with hybridization solution (2% BSA, 5x Denhardt’s solution, 4x SSC, 5% dextran sulphate, 35% deionized formamide, 10 U/ml RNase inhibitor) for 2 hours. The slides were then incubated overnight at room temperature in a humid chamber in the presence of 10 ng/µl Cy3 labelled oligonucleotide probes (Supplementary Table S3) in the aforementioned hybridization solution. Afterwards, slides were washed for 10 min, once with 4x SSC with 35% deionized formamide, followed by one wash each with 2x SSC and 1x SSC. Finally, slides were mounted with a mounting medium supplemented with 4’,6-diamino-2-phenylindole dihydrochloride (DAPI). Images were taken with an Olympus FluoView^TM^ FV1000 confocal microscope and analysed using Fluoview and ImageJ (NIH) software. FISH data were quantified as elsewhere described [71]. In brief, six cells per micrograph were selected randomly. Fluorescence intensities were measured using ImageJ with a plot profile analysis along a 3.5 µm line drawn across the nucleus with overhangs covering the cytoplasm. The results are expressed as the average value of relative fluorescence intensity ±SD.

### Quantification of nuclear division phenotypes

For DAPI counts, samples from FISH analysis were used. Nucleus (N) and kinetoplast (K) of 300 cells visualized by DAPI staining were counted for each cell line (uninduced *vs*. induced) and classified into five different categories (1K1N, 2K1N, 2K2N,1K0N,1K2N). The procyclic 29–13 strain was used as a control cell line. Numbers were plotted to the bar graphs. To determine the length of the nucleus, 100 cells were selected randomly. The length of the nucleus was measured using Image J by drawing an arbitrary line across the long axis of the nucleus.

### Flow cytometry

Approximately 3 × 10^6^ cells were pelleted and fixed in 1 ml of 70% methanol in 1xPBS and stored at 4°C overnight. Following a PBS wash, samples were incubated with 10 µg/ml Propidium iodide and 10 µg/ml of RNase A at 37°C for 45 min. Samples were analysed on a FACS Canto II (BD), collecting 50,000 gated events. Data were analysed using FlowJo v10.8 software.

### Annexin V/PI assay

To perform an Annexin V/PI assay for the TbNup144 and TbNup158 RNAi cell lines, we induced cell lines with 1 µg/ml of tetracycline for 28 h or 40 h according to cell line as indicated in the figure legends. For FACS analysis, approximately 5 × 10^6^ cells were harvested and washed in ice-cold 1xPBS and resuspended in 500 µl of 1x annexin-binding buffer (Invitrogen) containing 200 ng/ml of PI and 5 µl of FITC annexin V (Invitrogen). After 5 min of incubation, cells were analysed by flow cytometry on FACS Canto II (BD).

### Immunofluorescence assay

1 × 10^7^ cells were harvested and fixed to glass slides with 4% paraformaldehyde for 10 min. Cells were then permeabilized with 100% ice-cold methanol for 20 min and washed once with PBS. All subsequent incubation steps were performed in a humid chamber. To block non-specific binding sites, the slides were incubated with 5% milk in PBS supplemented with 0.05% Tween 20 for 45 min. Slides were then probed with primary mouse mAb62 antibodies (1:200 dilution), followed by extensive washes with PBS, and finally with secondary goat anti-mouse antibody coupled with AlexaFluor488. The slides were air-dried and mounted with mounting media containing DAPI (Invitrogen). Images were acquired on fluorescent microscope Zeiss Axioplan 2.

## Supplementary Material

Supplemental Material
