# Supplementary material for: Trafficking and/or division: Distinct roles of nucleoporins based on their location within the nuclear pore complex
Source: RNA Biol. 2022 May 1;19(1):650–61. doi: 10.1080/15476286.2022.2067711 (PMC9067531; doi:10.1080/15476286.2022.2067711)
Supplement: Supplemental Material [file KRNB_A_2067711_SM7683.docx]

**Supplementary Information**

**Trafficking and/or division: Distinct roles of nucleoporins based on their location within the nuclear pore complex**

Eva Hegedűsová^1^, Veronika Maršalová^1,2^, Sneha Kulkarni^1,2^ and Zdeněk Paris^1,2*^

**Supplementary Tables**

**Table S1.** Oligonucleotides used in this study (recognition sites for restriction endonucleases are underlined and the sequences from the ORF are highlighted with Bold)

| **Name** | **Sequence 5´ → 3´** |
| --- | --- |
| Nup62_F | CCCAAGCTT**GATCATGCTTACAATGCCAT** |
| Nup62_R | CGGGATCC**GTTCCAACAATTCGCTTAGA** |
| Nup53a_F | CCCAAGCTT**AAGATTACCGTGACTTTTCG** |
| Nup53a_R | CGGGATCC**CTAAGCGATCGTTGACTTCC** |
| Nup144_F | CCCAAGCTT**GTGTCTCTACGAGGTTAAGT** |
| Nup144_R | CGGGATCC**TATGGACCATCTACTGTGAC** |
| Nup158_F | CCCAAGCTT**GGCTACTGGTTTTGGTCAGC** |
| Nup158_R | CGGGATCC**GCAAACTCCGGAAGGTTCAG** |

**Table S2.** Oligonucleotides used for RT-PCR

| **Name** | **Sequence 5´ → 3´** |
| --- | --- |
| Nup62_F | ACTGGAGGATTTGGTAGTGG |
| Nup62_R | CACGGCACTTTGGCAATTGA |
| Nup53a_F | CAACTAACCCTGCCTCAACT |
| Nup53a_R | TCAAACTGCGCCAGTATGGT |
| Nup144_F | ACGAAACGCCCTCTCAAGAG |
| Nup144_R | ACGTCTCTCTTCAGGCTGTT |

**Table S3.** Oligonucleotides used for Northern blot hybridization and FISH.

| **Name** | **Sequence 5´ → 3´** |  |
| --- | --- | --- |
| tRNA^Tyr^ -3´exon | GTGGTCCTTCCGGCCGGAATCGAA |  |
| tRNA^Tyr^- intron | TGTGATACCTGCAAACTCTACAG |  |
| tRNA^Glu^ | TTCCGGTACCGGGAATCGAAC |  |
|  | **Sequence 5´ → 3´** | **Fluorophore** |
| TbtRNA^Tyr-mature^ | AACCAGCGACCCTGTGATCTAC | 5´end Cy3 |
| TbtRNA^Asp^ | CGGGTCACCCGCGTGACAGG | 5´ end Cy3 |
| TbtRNA^Phe^ | GCGACCCGGGATCGAACCAGGGACC | 5´ end Cy3 |

**Supplementary Figures**

**
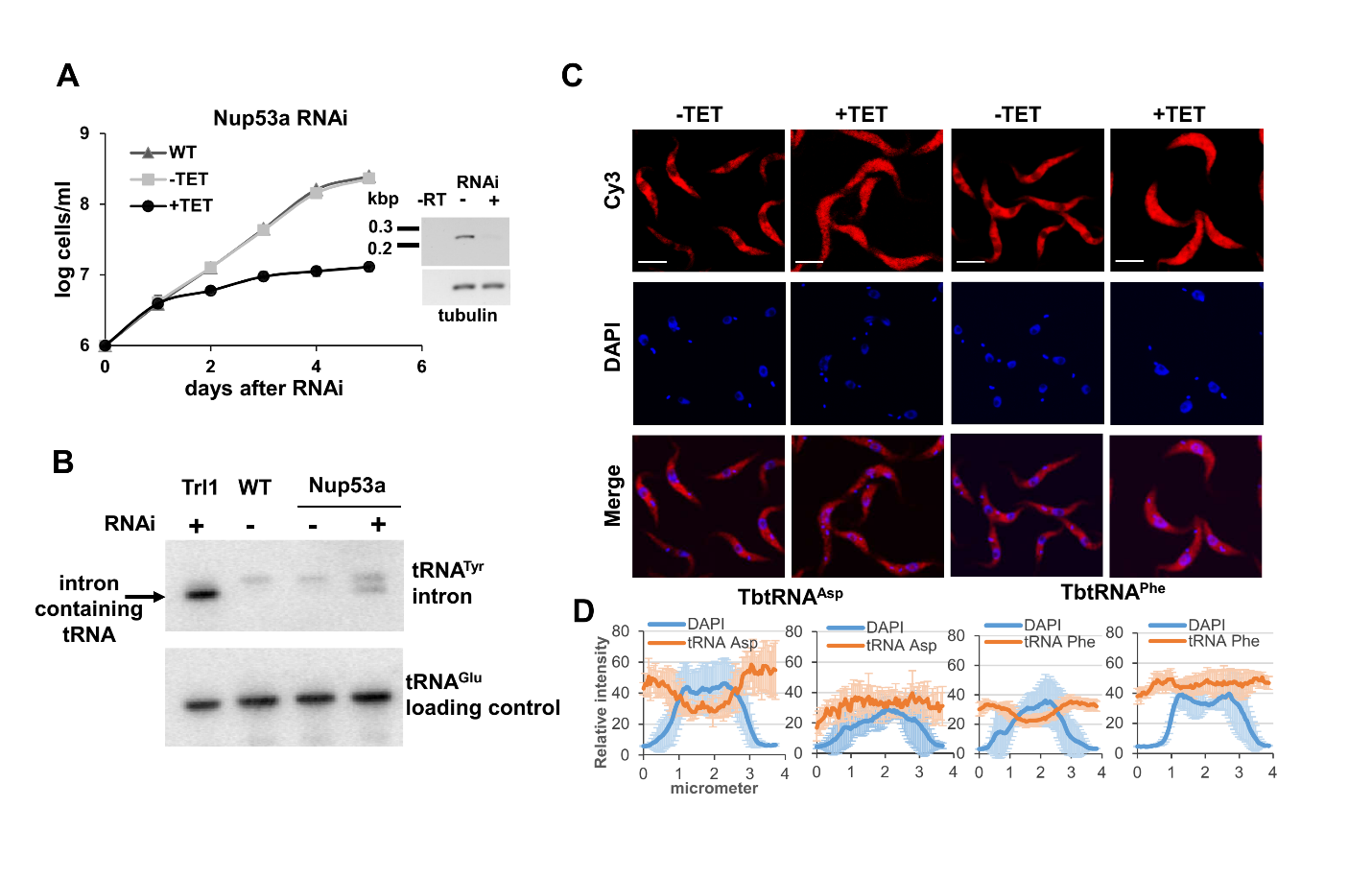
**

**Supplementary Figure S1.** Nucleoporin TbNup53a is essential for cell survival. **(A)** Growth curve of the procyclic form of *T. brucei,* wild-type (WT; triangle), non-induced (-TET; square), and RNAi-induced (+TET; circle) cell lines of TbNup53a. Three biological repeats were performed, and the average of three experiments was plotted, mean ±SD is shown. Inset: RT-PCR analysis showing the down-regulation of the mRNA levels and loading control. **(B)** Silencing of TbNup53a affects tRNA export of intron-containing tRNA^Tyr^. Total RNA was isolated from wild-type (WT), non-induced (-TET), and RNAi-induced (+TET) cells of TbNup53a and Northern hybridization was performed with a tRNA^Tyr^-intron probe to detect intron accumulation. Trl1 RNAi induced cells were used as a positive control. tRNA^Glu^ was used as a loading control for the experiment. The blots are representative of three independent experiments. **(C)** Silencing of nucleoporin TbNup53a affects nuclear tRNA export. To determine the subcellular localization of mature tRNA^Asp^ and tRNA^Phe^ in non-induced (-TET) and RNAi-induced 28 hr (+TET) cells, fluorescent *in situ* hybridization was performed. Micrographs show the subcellular localization of the mature tRNA^Asp^ and tRNA^Phe^ (red-Cy3). DAPI (blue) was used to stain the kinetoplast and nucleus DNA. Bars, 5 µm. **(D)** Quantification of the fluorescence intensity of tRNAs (orange) and DAPI (blue) in non-induced and 28 hr RNAi-induced TbNup53a cell line. Each graph shows the intensity profile of individual fluorophores (orange-tRNA^Phe,-Asp^, blue-DNA) of 6 randomly selected cells, representing the relative intensity average ±SD.


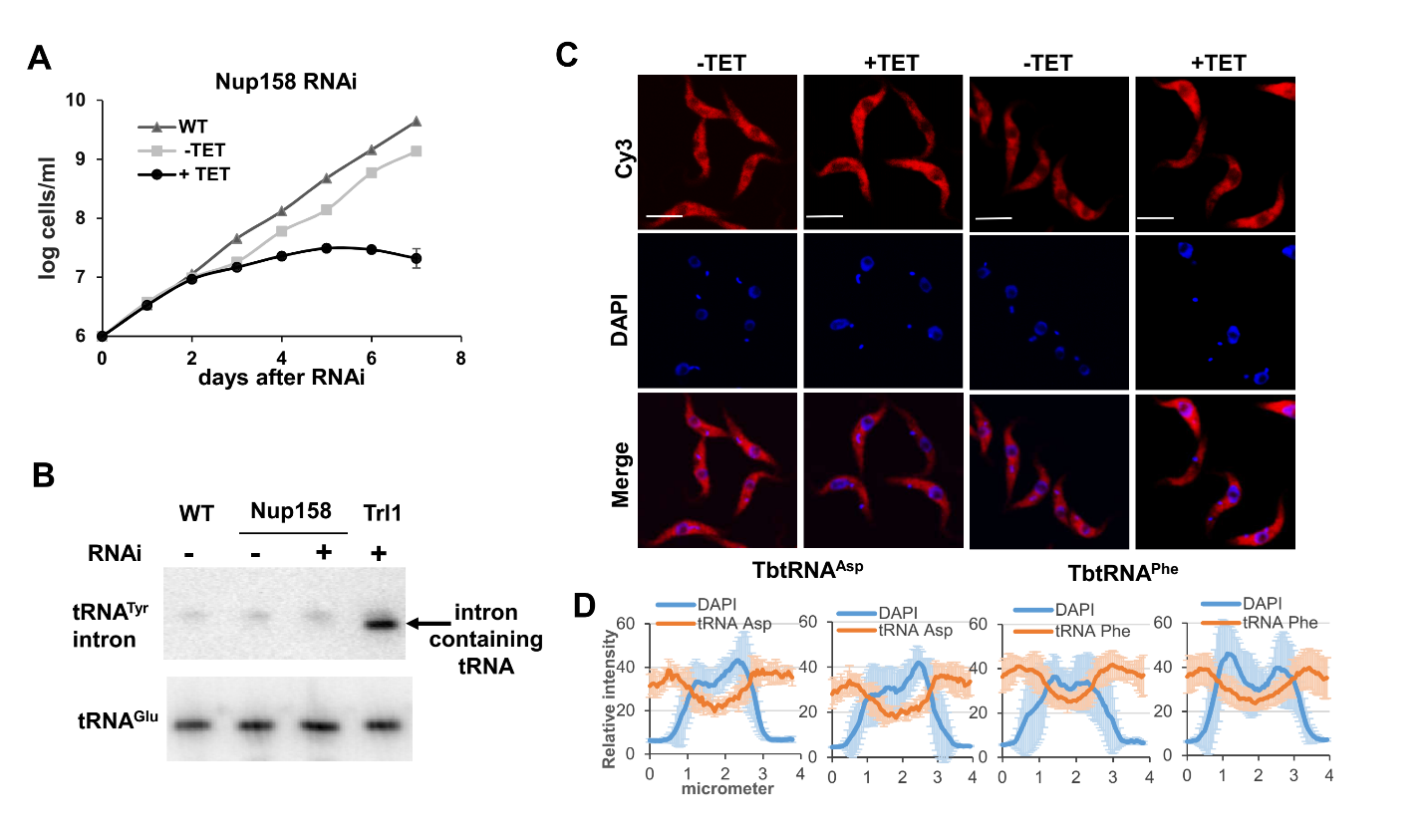


**Supplementary Figure S2.** Nucleoporin TbNup158 does not affect tRNA localization **(A)** Growth curve of the procyclic form of *T. brucei,* wild-type (WT; triangle), non-induced (-TET; square), and RNAi-induced (+TET; circle) cell lines of TbNup158. Three biological repeats were performed, and the average of three experiments was plotted, mean ±SD is shown. **(B)** Silencing of TbNup158 does not affect tRNA export of intron-containing tRNA^Tyr^. Total RNA was isolated from wild-type (WT), non-induced (-TET), and RNAi-induced (+TET) cells of TbNup158 and Northern hybridization was performed with a tRNA^Tyr^-intron probe to detect intron accumulation. Trl1 RNAi induced cells were used as a positive control. tRNA^Glu^ was used as a loading control for the experiment. The blots are representative of three independent experiments. **(C)** Silencing of nucleoporin TbNup158 does not affect mature tRNA export. To determine the subcellular localization of mature tRNA^Asp^ and tRNA^Phe^ in non-induced (-TET) and RNAi-induced 48 hr (+TET) cells, fluorescent *in situ* hybridization was performed. Micrographs show the subcellular localization of the mature tRNA^Asp^ and tRNA^Phe^ (red-Cy3). DAPI (blue) was used to stain the kinetoplast and nucleus DNA. Bars, 5 µm. **D)** Quantification of the fluorescence intensity of tRNAs (orange) and DAPI (blue) in non-induced and 48 hr RNAi-induced TbNup158 cell line. Each graph shows the intensity profile of individual fluorophores (orange-tRNA^Phe, -Asp^, blue-DNA) of 6 randomly selected cells, representing the relative intensity average ±SD.

**
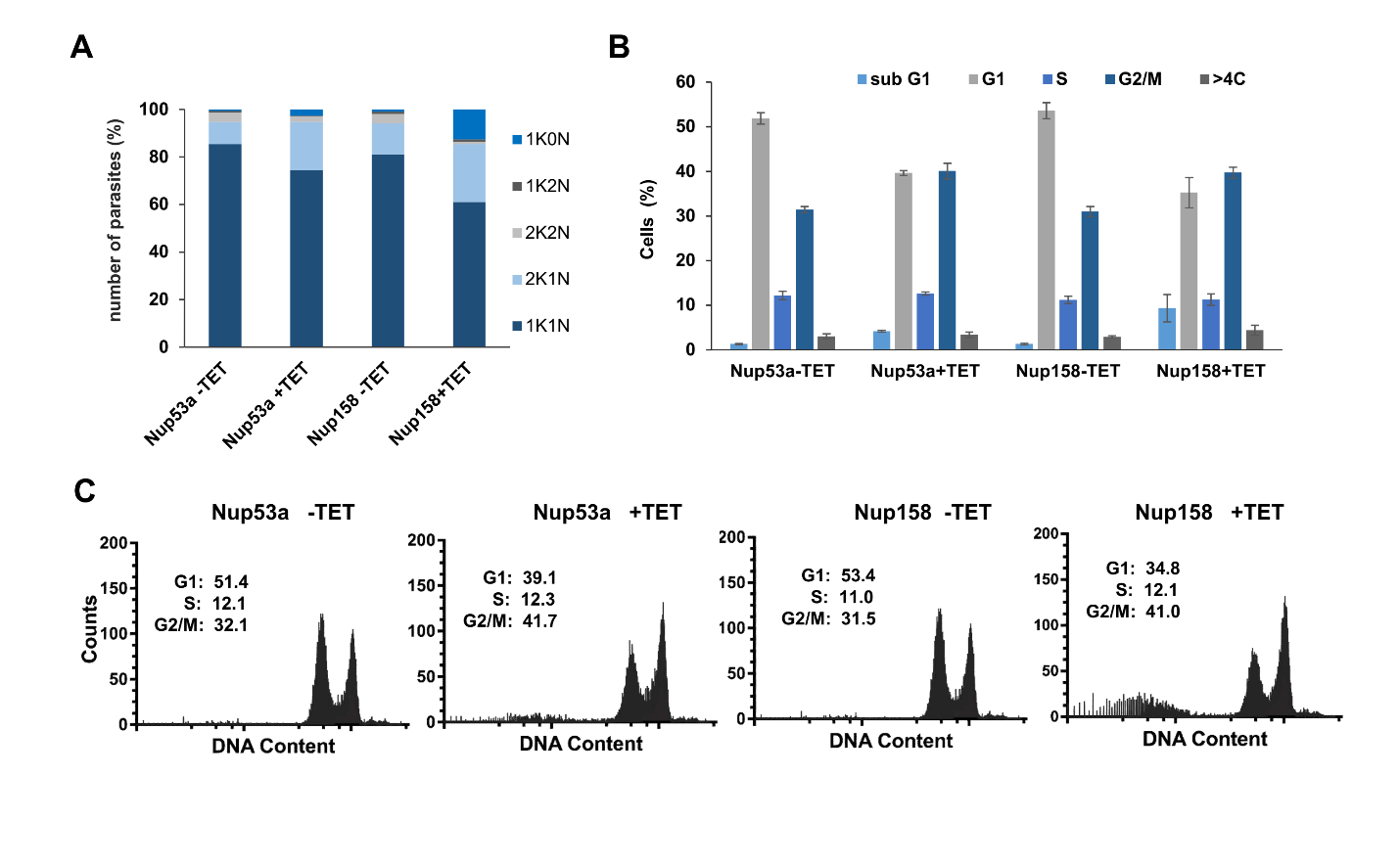
Supplementary Figure S3**. Silencing of TbNup158 causes difficulty in nuclei division resulting increased number of zoids compared with TbNup53a **A)** Quantification of nuclear (N) kinetoplast (K) ratio of non-induced (-TET) and RNAi-induced (+TET) TbNup53a (28 hr) and TbNup158 (40 hr) cells. 300 cells from each condition were counted to categorize the distinct stages of the cell cycle. TbNup158 RNAi-induced cells show an increased number of 2K1N cell types, suggesting slight arrest in the G2/M phase. **B)** Densitometry analysis of the cell cycle phases (sub-G1, G1, S, G2/M, >4C) for TbNup53a and TbNup158 RNAi cell lines. Mean scores from three biological replicates are plotted, and ±SD is shown. **C)** Flow cytometry profiles of non-induced, and RNAi-induced TbNup53a and TbNup158 cells. To determine the karyotype of the population, cells were fixed and stained with propidium iodide (indicating DNA content) (*x*-axes). Cell count is plotted on the *y*-axes. Downregulation of TbNup158 shows slight arrest in the G2/M phase (2K1N), suggesting that cell lines after induction encounter errors in completing mitosis and cytokinesis. Three biological repeats were performed, and representative histograms are shown. Inset: Percent of cells in G1, S and G2/M are shown.

**
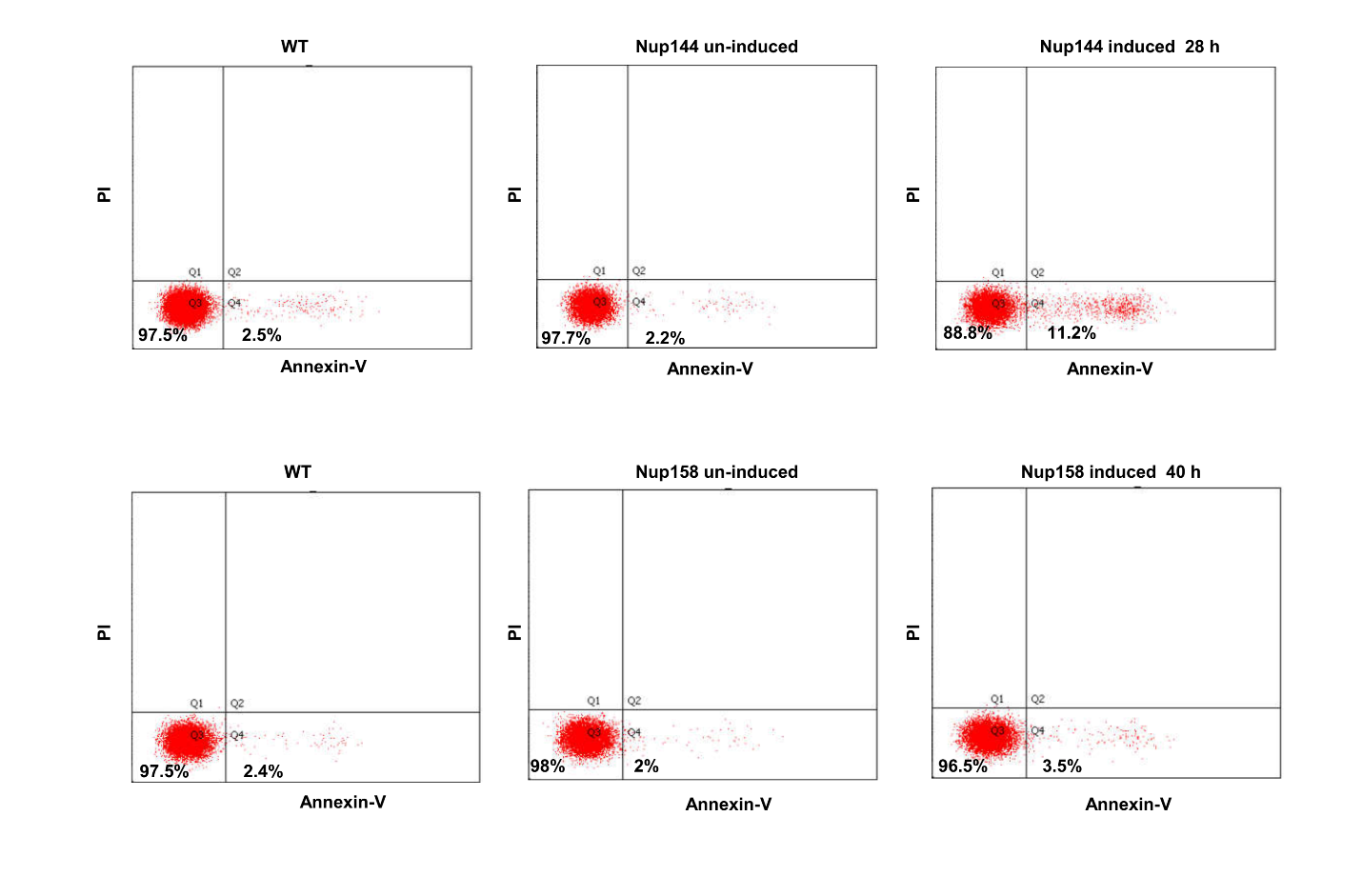
**

**Supplementary Figure S4.** Flow cytometry analysis by FITC Annexin V and propidium iodide staining revealed presence of early apoptotic cells of induced TbNup144 RNAi cell line. To determine the live, apoptotic or dead cells in the population, cells were stained with propidium iodide (*y*-axis) and annexin V (*x*-axis). **Top row:** Determination of cell population in parental (WT), un-induced and 28 hr induced TbNup144 RNAi cell line. Increased amount (11,2%) of early apoptotic cells were detected after 28 hr RNAi induction (quadrant Q4) compared with parental or un-induced cell lines (approx. 2.5%). **Bottom row:** WT, un-induced and 40 hr induced TbNup158 RNAi cell lines. The amount of early apoptotic cells (3,5%) in the case of induced TbNup158 is less pronounced compared with induced TbNup144. Three biological repeats were performed, and representative scatter plots are shown.
